# Regions of robust relative stability for PI controllers and LTI plants with unstructured multiplicative uncertainty: A second-order-based example

**DOI:** 10.1016/j.heliyon.2023.e18924

**Published:** 2023-08-06

**Authors:** Radek Matušů, Bilal Senol, Libor Pekař

**Affiliations:** a Centre for Security, Information and Advanced Technologies (CEBIA–Tech), Faculty of Applied Informatics, Tomas Bata University in Zlín, nám. T. G. Masaryka 5555, 760 01, Zlín, Czech Republic; b Software Engineering Department, Faculty of Engineering, Aksaray University, Bahçesaray Mahallesi, 68100, Aksaray, Turkey; c Department of Automation and Control Engineering, Faculty of Applied Informatics, Tomas Bata University in Zlín, nám. T. G. Masaryka 5555, 760 01, Zlín, Czech Republic; d Department of Technical Studies, College of Polytechnics Jihlava, Tolstého 1556, 586 01, Jihlava, Czech Republic

**Keywords:** Robust control, Robust relative stability, Robust performance, PI controllers, Unstructured multiplicative uncertainty, H-Infinity norm

## Abstract

This example-oriented article addresses the computation of regions of all robustly relatively stabilizing Proportional-Integral (PI) controllers under various robust stability margins *α* for Linear Time-Invariant (LTI) plants with unstructured multiplicative uncertainty, where the plant model with multiplicative uncertainty is built on the basis of the second-order plant with three uncertain parameters. The applied graphical method, adopted from the authors’ previous work, is grounded in finding the contour that is linked to the pairs of P–I coefficients marginally fulfilling the condition of robust relative stability expressed using the *H*_∞_ norm. The illustrative example in the current article emphasizes that the technique itself for plotting the boundary contour of robust relative stability needs to be combined with the precondition of the nominally stable feedback control system and with the line for which the integral parameter equals zero in order to get the final robust relative stability regions. The calculations of the robust relative stability regions for various robust stability margins *α* are followed by the demonstration of the control behavior for two selected controllers applied to a set of members from the family of plants.

## 1 Introduction

It may seem almost incredible that, despite the existence of many up-to-date advanced control approaches, Proportional-Integral-Derivative (PID) and especially Proportional-Integral (PI) controllers persistently represent the most widespread control strategy, widely employed not only in industrial automation but also in array of other aspects of our everyday lives [[1], [2], [3], [4], [5]]. Consequently, it is still worthwhile to investigate the PI(D)-based control systems, especially under various conditions of uncertainty [[6], [7], [8]].

From the viewpoint of robust control, the three predominant approaches to incorporating the uncertainty into a Linear Time-Invariant (LTI) model of a controlled system are parametric uncertainty [[9], [10], [11], [12]], unstructured uncertainty [11,[13], [14], [15], [16], [17]], and Linear Fractional Transformation (LFT) [14,[18], [19], [20], [21]]. Of these choices, the unstructured uncertainty-based method represents a reasonable balance between effectiveness and simplicity.

In an unstructured uncertainty model, the true structure of the plant (i.e., its order) may remain unknown, which means that not merely variations in parameters but sundry unmodeled complex dynamics as well may be encompassed in this model. On the other hand, the model of the plant remains relatively simple (first of all, it is still LTI), and it is suitable for control synthesis, especially for the methods based on the *H*_∞_ norm.

Some applications of robust control methods using *H*_∞_ techniques and models with unstructured uncertainty are presented in Refs. [[22], [23], [24]]. Nonetheless, the use of *H*_∞_ tools in control system synthesis frequently results in high-order and, hence, impractical controllers [25]. In spite of the attempts to restrict the order of *H*_∞_ controllers [[26], [27], [28]], many of these techniques are computationally very demanding. Consequently, several works naturally focused on various other approaches (using *H*_∞_ norms or not) to designing PI(D) controllers under unstructured uncertainty [15,17,25,[29], [30], [31], [32], [33], [34], [35], [36]].

Beyond a doubt, stability poses the most essential requirement for control systems. The notion of (absolute) stability can be broadened to relative stability [7,[37], [38], [39], [40]], which allows considering how far the system is from the border of (in)stability. Furthermore, relative stability is also an instrument for gaining a certain degree of performance because the shifted half-plane assures a given settling time [40]. In compliance with the same logic, the principle of robust stability can be broadened to robust relative stability, which entails that the prescribed relative stability is guaranteed for all possible plant family members, including the worst case.

This instance-oriented article focuses on the computation of regions of robust relative stability for PI controllers and LTI plants under unstructured multiplicative uncertainty. The plant model with multiplicative uncertainty is built on the basis of the second-order plant with three uncertain parameters, and the areas of robust relative stability are obtained in the plane of the controller parameters for various robust stability margins *α*. Subsequently, two controllers from various robust relative stability regions are chosen, and the corresponding control behaviors are verified for a set of representative members from the plant family. The thought of calculating the robust relative stability regions is adopted from Ref. [7]. In accordance with that work, a numerical procedure is used for obtaining the contours composed of the pairs of PI controller coefficients that marginally fulfill the specified condition of robust relative stability, defined through the *H*_∞_ norm. The numerical calculations of the contours are performed with some acceptable tolerance to the condition. In the previous papers, the boundary contour-based areas of robustly stabilizing PI controllers were applied to plants with multiplicative [15,35] and additive [33] uncertainty, but only for robust (absolute) stability. Then, the refinement of the technique for PID controllers, as well as the generalization of the method from the robust relative stability viewpoint, was provided in Ref. [7]. The current article emphasizes, among others, that the technique itself for plotting the boundary contour of robust relative stability needs to be combined with the precondition of the nominally stable closed-loop control system in order to get the final robust relative stability regions. Thus, the main contributions of this article can be seen in (1) the deeper elaboration of the technique for computation of regions of robust relative stability for PI controllers and LTI plants affected by unstructured multiplicative uncertainty, including the creation of the overall block diagram of the procedure, (2) plotting the robust relative stability regions in the P–I plane under various robust stability margins *α*, (3) emphasizing the importance of fulfilling the nominal stability condition, and (4) demonstration of the time domain control behavior for the selected controllers from various robust relative stability regions.

The rest of this article is structured as follows. The fundamental theoretical background, including the description of systems under unstructured multiplicative uncertainty, formulation of the robust relative stability condition, and calculation of robustly relatively stabilizing PI controllers, is recapped in Section 2. The same Section 2 also introduces an overall block diagram for a convenient practical application of the approach. Then, in Section 3, the specific plant model with the unstructured multiplicative uncertainty is created on the basis of the model with parametric uncertainty. Subsequently, the regions of all robustly relatively stabilizing PI controllers for various robust stability margins *α* are calculated in Section 4. Furthermore, Section 4 also demonstrates the examples of control behavior by means of two chosen controllers for various values of *α*. The final Section 5 offers some concluding remarks.

## 2 Theoretical background

### 2.1 Systems with unstructured multiplicative uncertainty

When compared with other uncertain models, the systems with unstructured uncertainty represent a convenient trade-off between effectiveness and simplicity. Further classification of the unstructured uncertainty models is available in Refs. [7,13,14,16]. Two basic sorts are multiplicative and additive uncertainty, and moreover, their inverse versions exist. When considering MIMO systems, a distinction must be made between the input and output variants of the multiplicative uncertainty (or its inverse alternative). Nevertheless, both these forms are equivalent for SISO systems, so the simple term multiplicative uncertainty will be used in this article.

Most likely, multiplicative uncertainty is the most frequently used kind of unstructured uncertainty. The perturbed model (transfer function) that contains the (input) multiplicative uncertainty is given as [7,14,16]:

(1) $$G_{M}\left(s\right)=\left[1+W_{M}\left(s\right)\Delta_{M}\left(s\right)\right]G_{0}\left(s\right)$$

where $G_{0}\left(s\right)$ symbolizes a nominal system transfer function, $W_{M}\left(s\right)$ stands for a weight function (usually considered as a stable and minimum-phase transfer function) representing the (frequency) distribution of the maximum magnitude of the uncertainty, and $\Delta_{M}\left(s\right)$ expresses the uncertainty itself.

The choice of a suitable weight function $W_{M}\left(s\right)$ must be made with respect to the inequality [7,14,16]:

(2) $$\left|\frac{G_{M}\left(j\omega \right)}{G_{0}\left(j\omega \right)}-1\right|\leq \left|W_{M}\left(j\omega \right)\right|\forall \omega$$

which says that, for all frequencies, the magnitude of the normalized perturbations (relative errors) of the model must be overlaid with the magnitude of $W_{M}\left(s\right)$.

The uncertainty $\Delta_{M}\left(s\right)$ can be represented by an arbitrary stable function that fulfills the inequality [7,14,16]:

$${\left\|\Delta_{M}\left(s\right)\right\|}_{\infty }\leq 1\;\Rightarrow \;\left|\Delta_{M}\left(j\omega \right)\right|\leq 1\forall \omega$$

A more detailed discussion on the stability condition for $\Delta_{M}\left(s\right)$, the graphical representation of (1), as well as information on other sorts of unstructured uncertainty, can be found in Ref. [7].

### 2.2 Robust relative stability under multiplicative uncertainty

Assume a complementary sensitivity function:

$$T_{0}\left(s\right)=\frac{L_{0}\left(s\right)}{1+L_{0}\left(s\right)}$$

where $L_{0}\left(s\right)$ is an open-loop system's transfer function:

$$L_{0}\left(s\right)=C\left(s\right)G_{0}\left(s\right)$$

and where C(s) and $G_{0}\left(s\right)$ is a controller and a nominal plant, respectively.

With respect to the classical robust control literature, the robust stability condition under consideration of multiplicative uncertainty can be formulated as follows: Under the assumption of a nominally stable feedback control system (that is, for $G_{0}\left(s\right)$), the related perturbed feedback control system (containing the plant affected by multiplicative uncertainty) is robustly stable if and only if [7,13,14]:

(3) $${\left\|W_{M}\left(s\right)T_{0}\left(s\right)\right\|}_{\infty }<1$$

Recently, the paper [7] has presented the relativized version of this condition as follows: Under the assumption of a nominally stable feedback control system, the related uncertain feedback control loop is robustly relatively stable, having a margin factor of *α* if and only if [7]:

(4) $${\left\|W_{M}\left(s\right)T_{0}\left(s\right)\right\|}_{\infty }<\frac{1}{\alpha }$$

Typically, the margin factor *α* is assumed to be positive. For the special case of α=1, both conditions (3) and (4) become identical.

More information on the robust stability, robust performance, and robust relative stability conditions, including their graphical interpretations and their versions for the other sorts of models under unstructured uncertainty, can be found in Ref. [7].

### 2.3 Calculation of robustly relatively stabilizing PI controllers

The key thought of the applied graphical approach [7] is grounded in depicting the contour that is defined by the pairs of P–I coefficients critically meeting the robust relative stability condition (4). Thus, the area of robustly relatively stabilizing coefficients is depictable in the plane of the controller parameters using the robust relative stability border P–I pairs, which means the couples of P–I coefficients that satisfy the condition [7]:

$${\left\|W_{M}\left(s\right)T_{0}\left(s\right)\right\|}_{\infty }=\frac{1}{\alpha }$$

However, one should be careful during practical calculations. As will be shown in the example below, although the corresponding P–I pairs define the curve(s) that separates the P–I plane into the parts where condition (4) is or is not fulfilled, these curves do not represent the true robust relative stability borders if the corresponding closed-loop systems are not nominally stable. In other words, we have to keep in mind the precondition as stated above the inequalities (4) and (3). Moreover, also the $k_{I}=0$ line has to be taken into consideration because it contributes to the division of the P–I plane into stable and unstable regions [40]. Then, the final region of robustly relatively stabilizing controllers can be obtained.

**Fig. 1:**
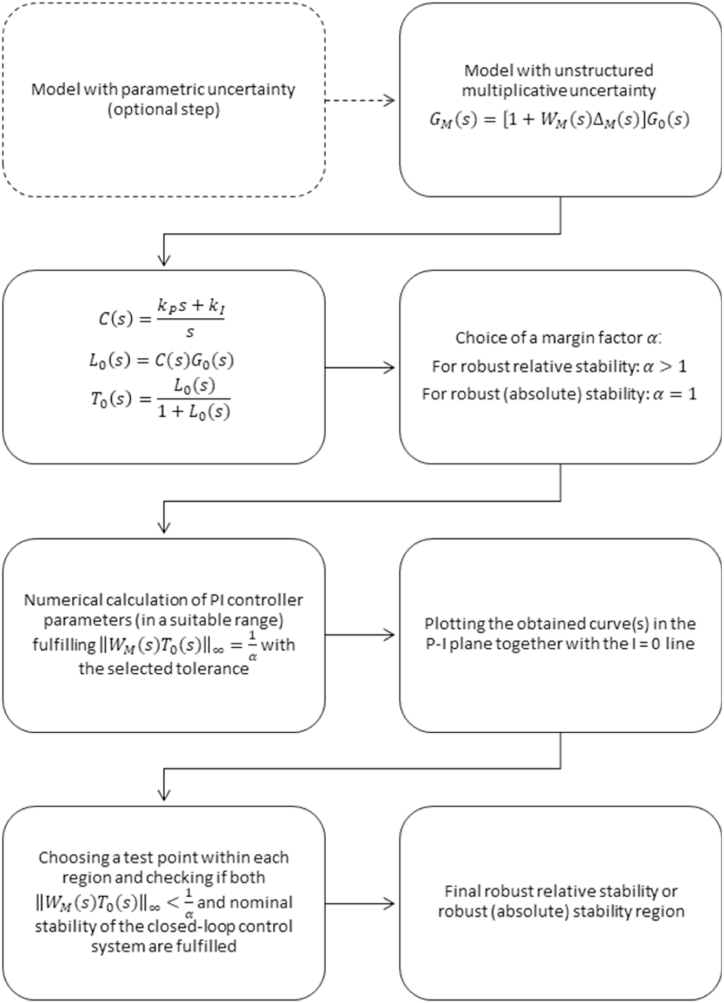
An overall block diagram for the procedure of obtaining the robust relative stability region for a PI controller and LTI plant with unstructured multiplicative uncertainty.

In order to facilitate the application of the proposed approach, an overall block diagram of the procedure is shown in Fig. 1.

The specific application of the described technique is shown by means of the following second-order-based example.

## 3 Uncertain mathematical models of the controlled plant

Assume that the controlled plant is given by a frequently used second-order transfer function with two different time constants, i.e.,:

(5) $$G_{par}\left(s,K,T_{1},T_{2}\right)=\frac{K}{\left(T_{1}s+1\right)\left(T_{2}s+1\right)}$$

where all three coefficients, namely gain *K*, the first time constant *T*_1_, and the second time constant *T*_2_, may vary within the following intervals [16]:

(6) $$\begin{array}{l} K\in \left[1.8,2.2\right]\text{,}\\ T_{1}\in \left[9,11\right]\text{,}\\ T_{2}\in \left[0.9,1.1\right] \end{array}$$

Since the applied method takes advantage of the models under unstructured multiplicative uncertainty (for the other modifications, see Ref. [7]), the original plant described by the parametrically uncertain model (5) must be substituted by the model with unstructured multiplicative uncertainty. Generally, not only variations of parameters but unmodeled dynamics as well may be hidden in the unstructured uncertainty; however, the model containing unstructured multiplicative uncertainty will be built “only” according to the model with parametric uncertainty in this example. Actually, this preliminary model with parametric uncertainty (5) is unnecessary from the viewpoint of this synthesis approach, but the model (5) is deliberately utilized here to demonstrate its connection with the model with unstructured multiplicative uncertainty (1). Several methods for the identification of models with parametric or unstructured uncertainty can be found, e.g., in Refs. [[41], [42], [43]].

In order to create the general model (1) (in this case, specific model (9), which was adopted from Ref. [16]), it is necessary to choose a nominal system transfer function *G*_0_(*s*) together with a weight function *W*_*M*_(*s*). First, the transfer function *G*_0_(*s*) was simply supposed to have the mean values of the uncertain parameters (6), that is:

(7) $$G_{0}\left(s\right)=\frac{2}{\left(10s+1\right)\left(s+1\right)}=\frac{0.2}{s^{2}+1.1s+0.1}$$

**Fig. 2:**
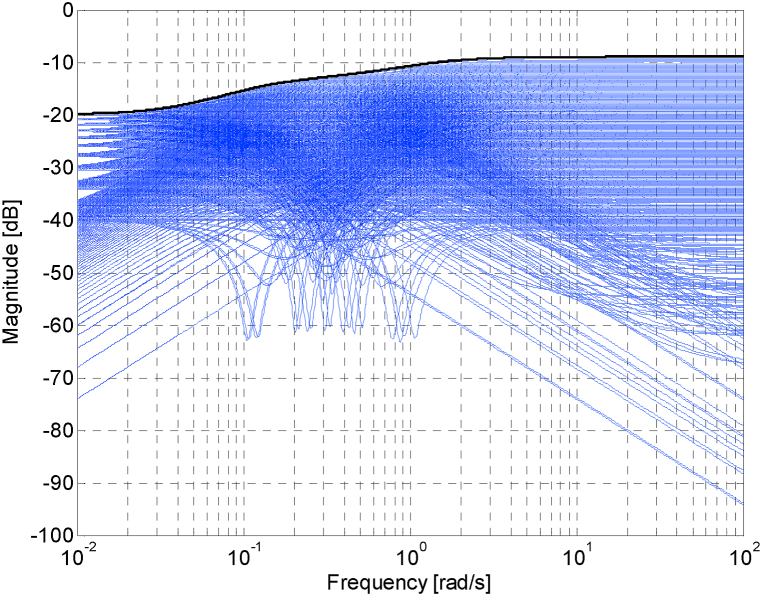
Bode magnitude diagram – the sampled set of normalized perturbations (blue curves) and weight function *W*_*M*_ (black curve) [16].

**Fig. 3:**
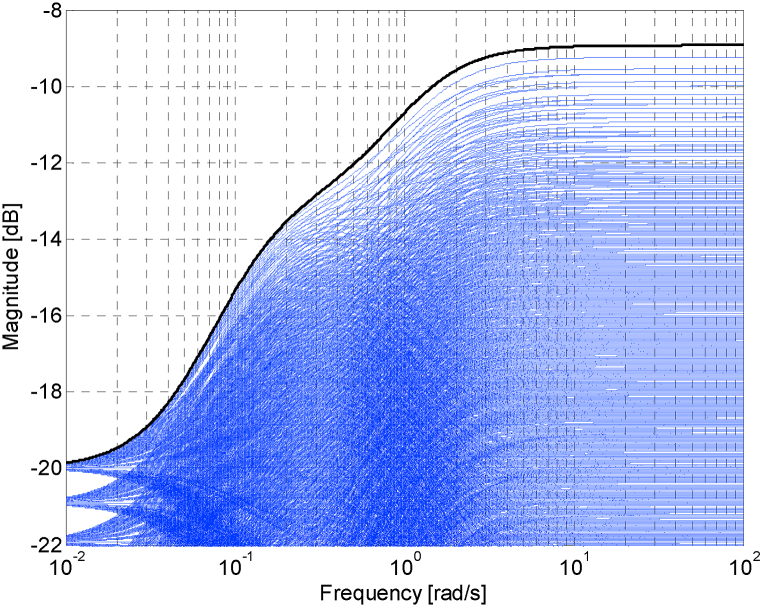
Zoomed version of the Bode magnitude diagram from Fig. 2 [16].

Then, the weight function *W*_*M*_(*s*), which must fulfill (2), was determined in Ref. [16] as the worst-case uncertainty member (with K=2.2, $T_{1}=9$, $T_{2}=0.9$) of the plant family (5). This worst-case combination of parameters tallies with the uppermost magnitude characteristics of the normalized perturbations from Fig. 2, Fig. 3 [16], and so the weight function was chosen exactly accordingly:

(8) $$W_{M}\left(s\right)=\frac{2.9s^{2}+2.2s+0.1}{8.1s^{2}+9.9s+1}$$

The Bode magnitude diagram for the weight function (8) (plotted with the black curve) and also for a sampled set of the relative perturbations $\left(G_{M}\left(s\right)/G_{0}\left(s\right)-1\right)$ for all combinations of parameters K, sampled from 1.8 to 2.2 with step 0.02, $T_{1}$, sampled from 9 to 11 with step 0.2, and $T_{2}$, sampled from 0.9 to 1.1 with step 0.02, (plotted with the blue curves) is depicted in Fig. 2 [16] and subsequently in its zoomed version in Fig. 3 [16].

The utilization of the nominal model (7) and the weight function (8) leads to the complete model with unstructured multiplicative uncertainty [16]:

(9) $$\begin{array}{l} G_{M}\left(s\right)=\left[1+W_{M}\left(s\right)\Delta_{M}\left(s\right)\right]G_{0}\left(s\right)\\ {\left\|\Delta_{M}\left(s\right)\right\|}_{\infty }\leq 1\\ G_{0}\left(s\right)=\frac{0.2}{s^{2}+1.1s+0.1}\\ W_{M}\left(s\right)=\frac{2.9s^{2}+2.2s+0.1}{8.1s^{2}+9.9s+1} \end{array}$$

The model (9) is more conservative than (5) because, as was shown in Ref. [16], although the weight function (8) exactly matches the worst-case normalized perturbation (see Fig. 3), the plant family (9) may still comprise some members which are not included in the parametric family (5) since the perturbations satisfying $\left|\Delta_{M}\left(j\omega \right)\right|\leq 1$ at all frequencies are supposed. The main purpose of $\Delta_{M}\left(s\right)$ is (except for acting as a scaling factor on the perturbation magnitude) to account for phase uncertainty [13]. Consequently, one should be aware of potential conservatism in the investigation of robust stability when a system with parametric uncertainty is modeled as a system with unstructured multiplicative uncertainty [16].

## 4 Robust relative stability regions

Assume that the closed-loop system is composed of the plant (9) and a PI controller:

(10) $$C\left(s\right)=\frac{k_{P}s+k_{I}}{s}$$

**Fig. 4:**
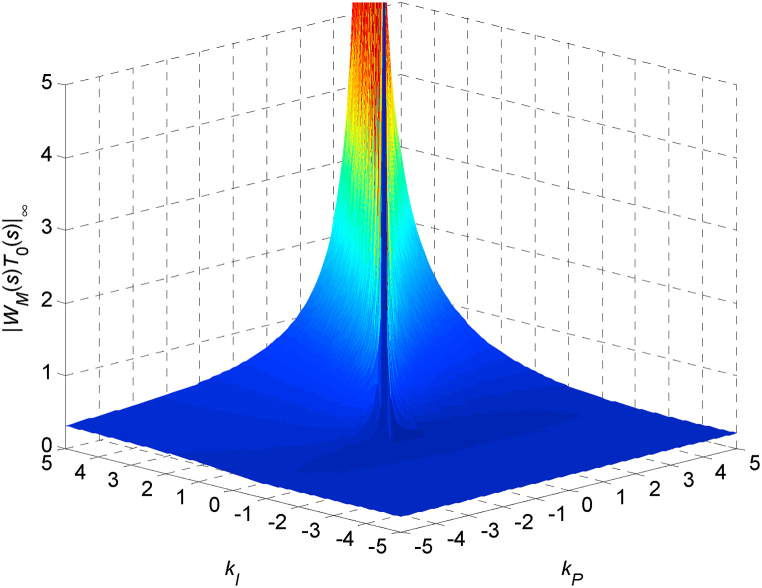
Dependence of ${\left\|W_{M}\left(s\right)T_{0}\left(s\right)\right\|}_{\infty }$ on controller's parameters *k*_*P*_ and *k*_*I*_

The value of ${\left\|W_{M}\left(s\right)T_{0}\left(s\right)\right\|}_{\infty }$ in the robust stability condition (3) and robust relative stability condition (4) depends on the parameters of the controller (10). Fig. 4 indicates this dependence for a selected range of parameters *k*_*P*_ and *k*_*I*_ (from −5 to 5 with step 0.1).

**Fig. 5:**
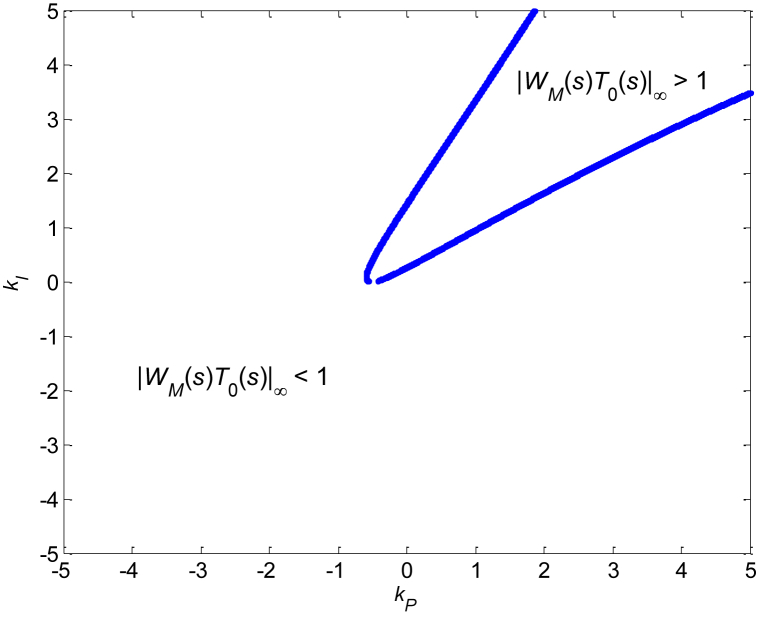
Contour ${\left\|W_{M}\left(s\right)T_{0}\left(s\right)\right\|}_{\infty }=1$ plotted in the P–I plane (under the same range of controller's parameters as in 3D Fig. 4).

The 2D contour ${\left\|W_{M}\left(s\right)T_{0}\left(s\right)\right\|}_{\infty }=1$, which can be obtained from the 3D graph in Fig. 4 and which represents the critical value from the viewpoint of (absolute) robust stability, is depicted in Fig. 5. It was calculated numerically by sampling the parameters *k*_*P*_ and *k*_*I*_ and computing the points that comply with the condition:

(11) $$\left|{\left\|W_{M}\left(s\right)T_{0}\left(s\right)\right\|}_{\infty }-1\right|<0.001$$

which means that the points fulfilling ${\left\|W_{M}\left(s\right)T_{0}\left(s\right)\right\|}_{\infty }=1$ with the selected tolerance of one per mille form the contour in Fig. 5. The step for sampling both controller parameters was chosen as 0.001, and the range of parameters for imaging is intentionally the same as in Fig. 4, i.e., from −5 to 5 for both *k*_*P*_ and *k*_*I*_.

**Fig. 6:**
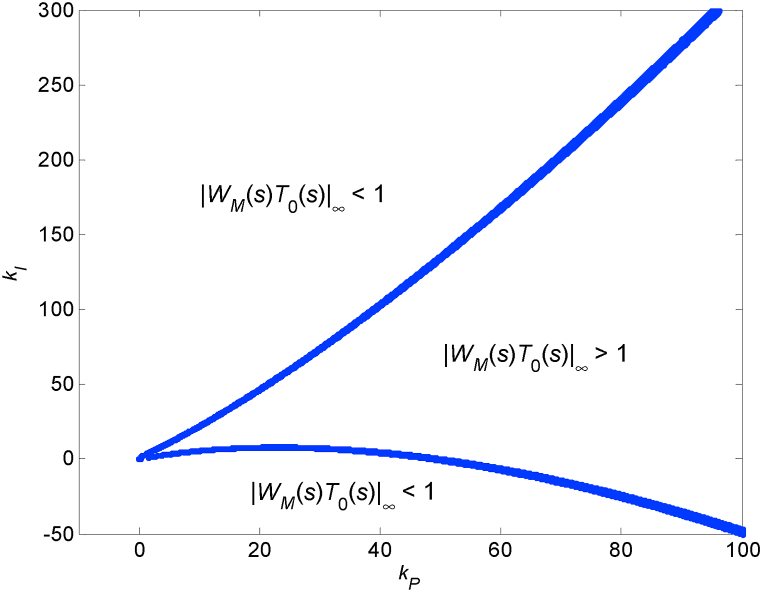
Contour ${\left\|W_{M}\left(s\right)T_{0}\left(s\right)\right\|}_{\infty }=1$ plotted in the P–I plane (under a wider range of controller's parameters).

The contour in Fig. 5 divides the displayed part of the P–I plane into the smaller inner area where ${\left\|W_{M}\left(s\right)T_{0}\left(s\right)\right\|}_{\infty }>1$ and the bigger outer area where ${\left\|W_{M}\left(s\right)T_{0}\left(s\right)\right\|}_{\infty }<1$. However, since the areas are open, a wider perspective is needed in order to have a more complete knowledge of their shape. Thus, such a more distant view (for *k*_*P*_ up to 100 and *k*_*I*_ up to 300) is shown in Fig. 6. In this case, the sampling step for parameters is 0.1 and a chosen numerical tolerance for fulfilling the robust stability condition is one per cent, which means condition (11) changes to:

$$\left|{\left\|W_{M}\left(s\right)T_{0}\left(s\right)\right\|}_{\infty }-1\right|<0.01$$

The first look at Fig. 4, Fig. 5, and especially 6 may give the impression that the robust stability region is represented by a large open area above the upper, under the lower, and left from both parts of the ${\left\|W_{M}\left(s\right)T_{0}\left(s\right)\right\|}_{\infty }=1$ contour. Nevertheless, this is “only” the area of ${\left\|W_{M}\left(s\right)T_{0}\left(s\right)\right\|}_{\infty }<1$, and the determination of the true robust stability region needs a more careful inspection.

**Fig. 7:**
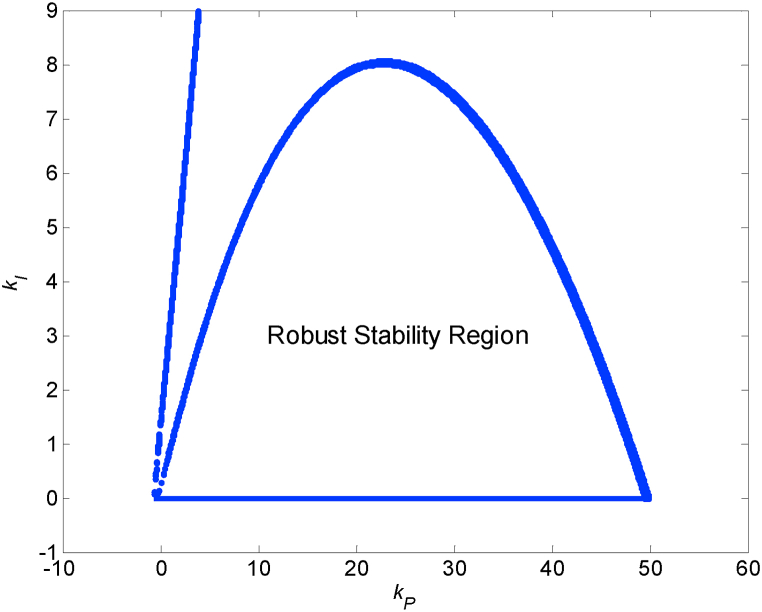
Robust stability region for the plant (9).

Actually, the upper part of the ${\left\|W_{M}\left(s\right)T_{0}\left(s\right)\right\|}_{\infty }=1$ contour (above the virtual $k_{I}=0$ line) has no practical meaning from the robust stability point of view because the prerequisite of the nominally stable feedback control system is not fulfilled above this part of the curve (see precondition before equation (3)). On the other hand, the lower part of the contour is significant and demarcates the robust stability border, but not the whole area below it represents the robust stability region because the actual region of robust stability is influenced by $k_{I}=0$ line as well (in this specific case, negative *k*_*I*_ makes the control system unstable). Accordingly, the region of (absolutely) robustly stabilizing PI regulators for the controlled plant under unstructured multiplicative uncertainty (9) is depicted in Fig. 7. The parameters of the applied numerical procedure were modified as follows: the sampling steps for both *k*_*P*_ and *k*_*I*_ were 0.01 and the robust stability condition tolerance was two per mille, i.e., the inequality (11) changes to:

$$\left|{\left\|W_{M}\left(s\right)T_{0}\left(s\right)\right\|}_{\infty }-1\right|<0.002$$

The region of robust stability in Fig. 7 represents the area inside which all possible combinations of the parameters *k*_*P*_ and *k*_*I*_ ensure robust stability of the feedback control loop consisting of the specific PI regulator and the controlled system model under unstructured multiplicative uncertainty (9).

**Fig. 8:**
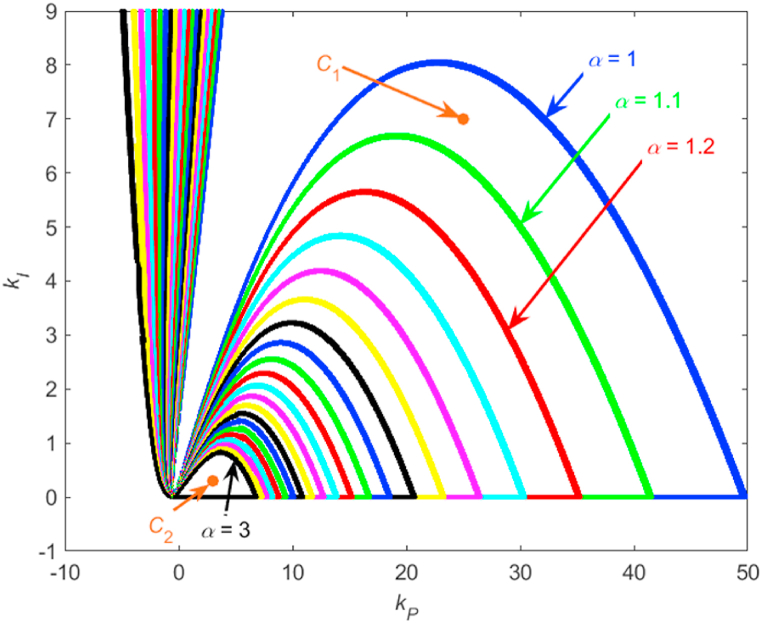
A set of robust relative stability regions for *α* from 1 to 3 with step 0.1 for the plant (9) with marked positions of controllers *C*_1_(*s*) (12) and *C*_2_(*s*) (13).

In the next phase, attention will be paid to obtaining the regions of robustly relatively stabilizing PI controllers. Generally, the calculation procedure and its parameters (sampling of *k*_*P*_ and *k*_*I*_ as well as the tolerance) stay identical to the previous case of robustly (absolutely) stabilizing controllers, however the conditional inequality (3) is modified to (4) by adding a margin factor *α*. Thus, the numerical procedure is based on the condition:

$$\left|{\left\|W_{M}\left(s\right)T_{0}\left(s\right)\right\|}_{\infty }-\frac{1}{\alpha }\right|<0.002$$

where the margin factor *α* can be chosen. A set of 21 robust relative stability regions for *α* from 1 to 3 with step 0.1 is plotted in Fig. 8. The first value α=1 corresponds to the special case of robust (absolute) stability, and thus it concurs with the region from Fig. 7. As can be seen, the area of robust relative stability region decreases with increasing size of *α*.

In order to demonstrate the examples of control behavior for the robust relative stability regions from Fig. 8, two controllers, *C*_1_(*s*) and *C*_2_(*s*), are chosen:

(12) $$C_{1}\left(s\right)=\frac{25s+7}{s}$$

(13) $$C_{2}\left(s\right)=\frac{3s+0.3}{s}$$

Their positions in the P–I plane are indicated in Fig. 8, which shows that controller *C*_1_(*s*) (12) is located in the robust relative stability region corresponding to the value of robust stability margin *α* between 1 and 1.1, and controller *C*_2_(*s*) (13) is placed in the robust relative stability region for much higher *α*. This means that both controllers guarantee robust relative stabilization of the plant (9) but *C*_2_(*s*) assures a higher robust stability margin and, consequently, also a higher level of robust performance when compared to *C*_1_(*s*). This will be demonstrated by means of the time domain control simulations.

Despite the fact that the computation of robust relative stability regions was grounded in the model containing unstructured multiplicative uncertainty (9), the control simulations utilize the (less conservative [16]) model with parametric uncertainty (5) for simplicity. More specifically, three uncertain parameters (6) of system (5) are sampled, namely, K=1.8:0.1:2.2, $T_{1}=9:0.5:11$ and $T_{2}=0.9:0.05:1.1$, which means that there are $5^{3}=125$ representative members of the plant family (5) used in simulations. The simulation time is 30 s, and the reference signal is represented by the unit step.

**Fig. 9:**
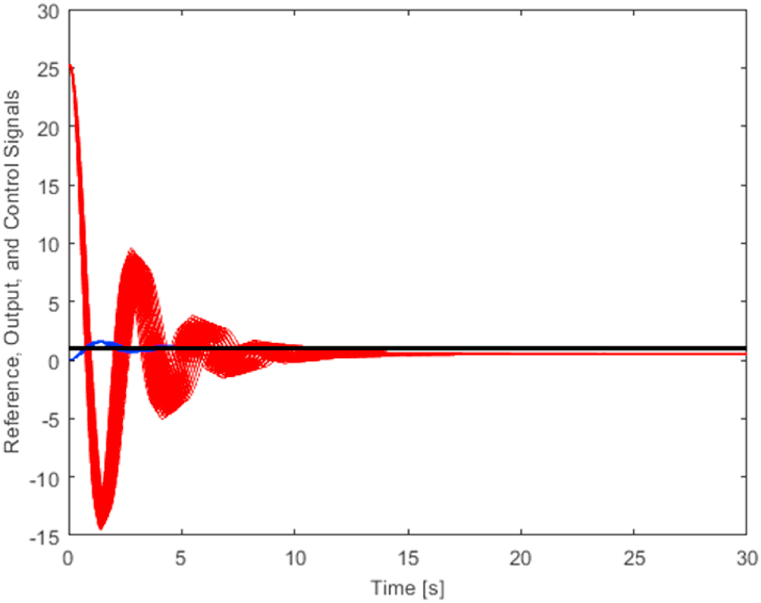
Control of 125 representatives of the plant family (5) using controller *C*_1_(*s*) (12) – the full view (blue curves – output signals, red curves – control signals).

**Fig. 10:**
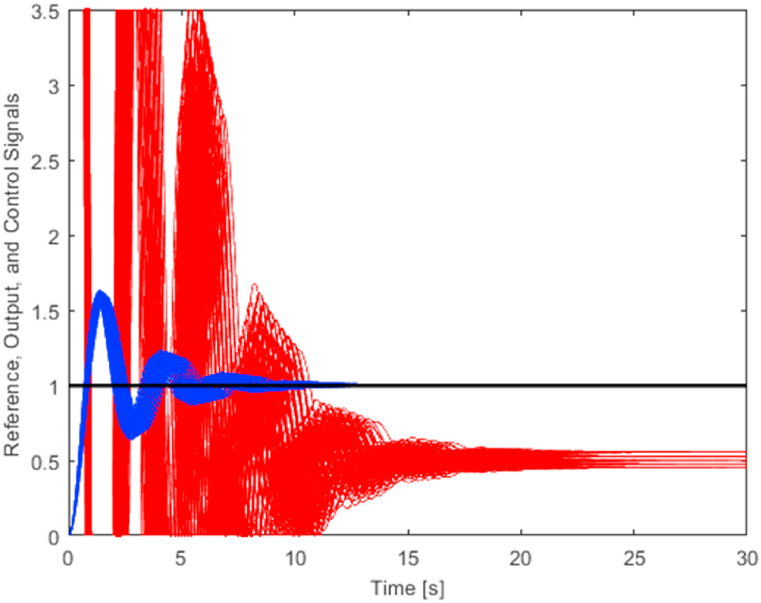
Control of 125 representatives of the plant family (5) using controller *C*_1_(*s*) (12) – a zoomed view (blue curves – output signals, red curves – control signals).

Fig. 9 presents a set of 125 output signals (plotted with the blue curves) and a set of 125 related control signals (plotted by the red curves) for controller *C*_1_(*s*) (12). The black line determines the reference signal. In order to make the output signals more visible, a zoomed version of Fig. 9 with the narrower perspective for the vertical axis is provided in Fig. 10.

**Fig. 11:**
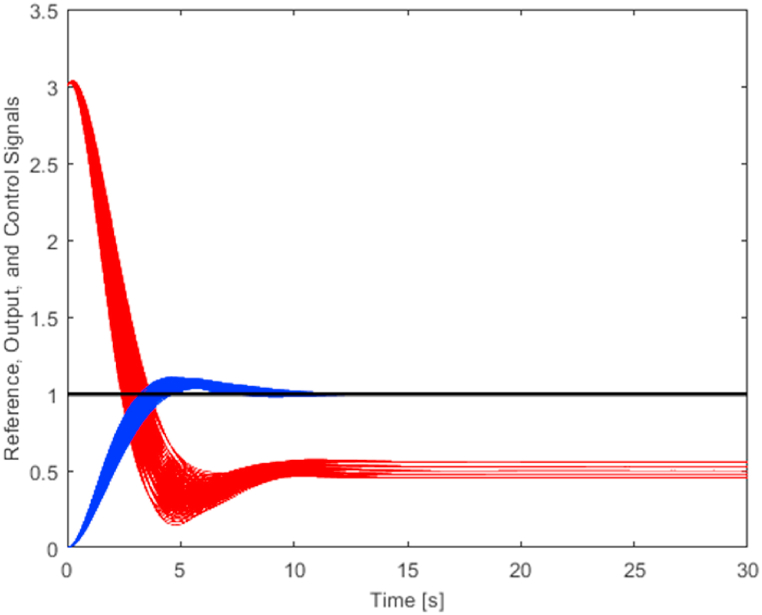
Control of 125 representatives of the plant family (5) using controller *C*_2_(*s*) (13) (blue curves – output signals, red curves – control signals).

Then, analogous control simulations are accomplished for controller *C*_2_(*s*). As in the previous Fig. 9, Fig. 10, the blue and red curves in Fig. 11 represent 125 output and 125 control signals, respectively.

As can be seen from the time domain control simulations, both controllers really assure robust relative stability (i.e., relative stability for all members of the uncertain model, including the worst case) and, as expected, *C*_2_(*s*) provides a higher robust stability margin, which also means a higher degree of robust performance when compared to *C*_1_(*s*). However, it should be emphasized that the applied controllers represent only examples of the potential choice since the presented technique itself does not lead to any specific controller. The aim of the method is to find all PI controllers satisfying the robust relative stability condition for a selected robust stability margin *α*. The final choice of the controller would depend on the specific requirements for a given application. An illustration of the practical laboratory application of the approach using the physical model of an air-heating tunnel can be found in Refs. [7,44].

## 5 Conclusions

The article dealt with obtaining the areas of all robustly relatively stabilizing PI controllers under various robust stability margins *α* for LTI systems with unstructured multiplicative uncertainty, whose mathematical model is built on the basis of the second-order plant with parametric uncertainty. The used numerical-graphical *H*_∞_ norm-based method is adopted from the authors’ previous work. The presented illustrative example stresses that the graphical technique itself needs to be combined with the precondition of the nominally stable feedback control system in order to obtain the valid final robust relative stability regions.

The advantage of the approach is the relatively simple computation of robust relative stability regions in the plane of PI controller parameters for LTI plants under unstructured multiplicative uncertainty with a user choice of the robust stability margin *α*, and its possible easy modification for other types of unstructured uncertainties [7]. On the contrary, the choice of the ranges for coefficients *k*_*P*_ and *k*_*I*_ and the choice of the tolerance for numerical solving the robust relative stability condition are not straightforward. Moreover, the outcome of the method is not any specific controller but the region of all robustly relatively (or only robustly absolutely, in the event of α=1) stabilizing PI controllers. Potential future research could be aimed at the extension of the technique to fractional-order controllers.

## Author contribution statement

Radek Matušů;: Conceived and designed the experiments; Performed the experiments; Analyzed and interpreted the data; Contributed reagents, materials, analysis tools or data; Wrote the paper.

Bilal Senol, Libor Pekař: Contributed reagents, materials, analysis tools or data; Wrote the paper.

Conceived and designed the experiments; Performed the experiments; Analyzed and interpreted the data; Contributed reagents, materials, analysis tools or data; Wrote the paper.

## Data availability statement

Data included in article/supplementary material/referenced in article.

## Declaration of competing interest

The authors declare that they have no known competing financial interests or personal relationships that could have appeared to influence the work reported in this paper.
